# Effect of pre-incisional infiltration with bupivacaine liposome for postoperative pain in patients undergoing acoustic neuroma surgery: study protocol for a prospective, double-blind, randomized controlled study

**DOI:** 10.3389/fmed.2025.1661276

**Published:** 2025-10-09

**Authors:** Maolin Ran, Ailing Song, Xiaochen Liu, Yu Zhou, Feng Chen, Qin Cui, Hongjiao Xu, Jinbao Li

**Affiliations:** Department of Anesthesiology, Shanghai General Hospital, Shanghai Jiao Tong University School of Medicine, Shanghai, China

**Keywords:** pre-incisional infiltration, liposomal bupivacaine, ropivacaine, acoustic neuroma surgery, postoperative pain

## Abstract

**Background:**

Post-craniotomy pain, relatively common in neurosurgery, is often inadequately managed. Preincisional infiltration with ropivacaine provides effective analgesia for post-craniotomy pain, although its duration of action is limited. Liposomal bupivacaine, a long-acting local anesthetic, can provide analgesia for up to 72 h. However, there is a paucity of research on its efficacy in post-craniotomy analgesia. This study hypothesizes that pre-incisional infiltration with liposomal bupivacaine will demonstrate superior analgesic efficacy compared with ropivacaine in patients undergoing acoustic neuroma surgery.

**Methods:**

This single-center, double-blind, randomized controlled study will recruit 112 patients scheduled to undergo acoustic neuroma surgery. We will compare the effects of liposomal bupivacaine and ropivacaine on postoperative pain when administered via preincisional infiltration before surgery. The primary outcome is the pain score at 24 h postoperatively. Secondary outcomes include the incidence of postoperative nausea and vomiting, amount of postoperative analgesic consumption, changes in vital signs before and after skin incision, and postoperative recovery scale.

**Discussion:**

This randomized controlled trial aims to evaluate the superior effects of pre-incisional infiltration of liposomal bupivacaine on postoperative pain control in patients undergoing acoustic neuroma surgery. This may provide a more effective analgesic regimen for patients undergoing craniotomies.

## 1 Introduction

Treatment for craniocerebral injury or intracranial space-occupying lesions often involves open-cranial surgery, an invasive procedure that inevitably causes pain that is not effectively controlled (1). Many reports have indicated that open-cranial surgery stimulates the scalp, including the soft tissue, muscles, and dura mater, causing varying degrees of pain in patients during the perioperative period, both during and after surgery. Over 60% of patients undergoing open-cranial surgery experience moderate to severe postoperative pain, with the most significant pain occurring between 24–48 h after surgery (2). Furthermore, postoperative pain may have long-term effects on patients. Research has indicated that up to 32% of patients who undergo open-cranial surgery develop chronic pain originating from acute pain. Additionally, 36.7%–49.5% of patients experience persistent pain for up to 12 weeks after surgery; 33%–43% experience persistent pain for more than 1 year; and 28.4% experience persistent pain for more than 3 years (3–5). This phenomenon is primarily attributed to failure to manage acute pain promptly and effectively. Chronic pain can alter the neuroendocrine system, impair postoperative cognitive function, and lead to long-term anxiety. These factors may further result in non-compliance with clinical treatment, thereby adversely affecting patient recovery (6, 7). Effective postoperative analgesia significantly reduces the incidence of complications and lowers disability and mortality rates. Therefore, effective control of pain during and after open-cranial surgery is of great significance for the surgery and prognosis of such patients (8–11). Effective pain control is an essential component of perioperative brain health strategies.

Acoustic neuroma, also known as a vestibular schwannoma, is a common neurosurgical condition requiring open-cranial surgery. It accounts for approximately 5% of intracranial tumors (12). Given the presence of many critical neural structures near the tumor site, such as the facial nerve, monitoring of neural function is typically required during surgery. This ensures that the tumor is removed while preserving facial nerve function, thereby reducing the incidence of postoperative facial paralysis (13–15). However, intraoperative neural function monitoring restricts the use of muscle relaxants and inhalation anesthetics, posing challenges in anesthetic management (16, 17). To avoid adverse situations, such as pain responses and intraoperative awareness, anesthesiologists often administer higher doses of opioid drugs to compensate for the lack of anesthetic depth. However, excessive opioid use can lead to several complications, including respiratory depression, postoperative nausea and vomiting (PONV), hypercapnia, cerebral vasodilation, increased intracranial pressure, and excessive sedation during the recovery and early postoperative periods (18). Given that patients with acoustic neuromas are already at a high risk of PONV, the use of higher opioid doses further increases the incidence of these adverse reactions (19–21). The use of opioid drugs postoperatively can interfere with early neurological examinations. Additionally, severe pain may necessitate their use as rescue rather than routine analgesics. Therefore, to avoid adverse reactions, patients with acoustic neuromas are not advised to receive intravenous postoperative analgesia. The commonly preferred methods are scalp nerve block and local incisional infiltration. However, a scalp nerve block may cause temporary facial nerve paralysis postoperatively, which is not conducive for intraoperative neural monitoring or early neurological assessment of surgical outcomes. This can directly delay the surgeon’s ability to judge the patient’s condition and may negatively affect recovery (22, 23). Therefore, optimal local incisional infiltration is considered the most favorable pain management method for patients undergoing acoustic neuroma surgery (24). Ropivacaine and bupivacaine are commonly used as local anesthetics for local incisional infiltration in clinical practice. Ropivacaine is an amide-type local anesthetic that is known for its good analgesic effects, minimal adverse reactions, and low cardiac toxicity. However, its duration of action is relatively short (2–6 h), which is insufficient to cover the patient’s entire pain period (25). Bupivacaine is a widely used long-acting local anesthetic with a duration of action of 6–8 h. However, it has higher cardiac toxicity, and its duration of action is insufficient to meet the clinical needs for postoperative analgesia (26). In 2011, the U.S. Food and Drug Administration approved a multilayered, foam-based bupivacaine liposome injection (trade name: Exparel) based on Depo Foam™ technology (27). This formulation encapsulates local anesthetics in a drug delivery system to provide long-lasting analgesia for surgical patients, with effects lasting up to 72 h (28). Bupivacaine liposomes can significantly improve postoperative analgesia in orthopedic, gynecological, abdominal, and plastic surgeries and are safe (29–31).

Pain associated with systemic hypertension, anxiety, and vomiting can also lead to intracranial hypertension. This condition is difficult to distinguish from postoperative neurosurgical complications and may mask or exacerbate related symptoms. Poor pain control progressing to chronic pain can lead to long-term mental anxiety and tension, potentially affecting cognitive function and hindering recovery. Effective postoperative analgesia can reduce the incidence of adverse reactions and complications following surgery. Bupivacaine liposome, a new local anesthetic, has a duration of action of up to 72 h. Administering it via local infiltration at the incision site before surgery can provide analgesia that covers the pain following open-cranial surgery, potentially providing better postoperative analgesia, reducing postoperative complications, and promoting early recovery. A recent meta-analysis by Fiore et al., the largest to date on craniotomy analgesia, provides high-certainty evidence that non-steroidal anti-inflammatory drugs and acetaminophen reduce pain 24 h postoperatively and that ropivacaine scalp block provides effective analgesia within the first 6 h after surgery (32). However, its limited duration of action fails to cover the peak pain period occurring 24–48 h post-craniotomy (2, 6). Therefore, our study seeks to build upon this foundation by evaluating whether pre-incisional infiltration with liposomal bupivacaine, which can provide analgesia for up to 72 h (33), offers superior and sustained pain control compared with ropivacaine, potentially bridging this critical analgesic gap.

This study aims to compare the effects of ropivacaine and liposomal bupivacaine for pre-incisional local infiltration on perioperative pain in patients undergoing acoustic neuroma surgery. Additionally, this study will evaluate opioid and other analgesic consumption, postoperative hospital stay, recovery, and the incidence of postoperative complications between the two groups. This study aims to provide a reference for optimizing anesthetic and analgesic regimens in patients with acoustic neuroma during the perioperative period.

## 2 Methods and analysis

### 2.1 Study design

**FIGURE 1:**
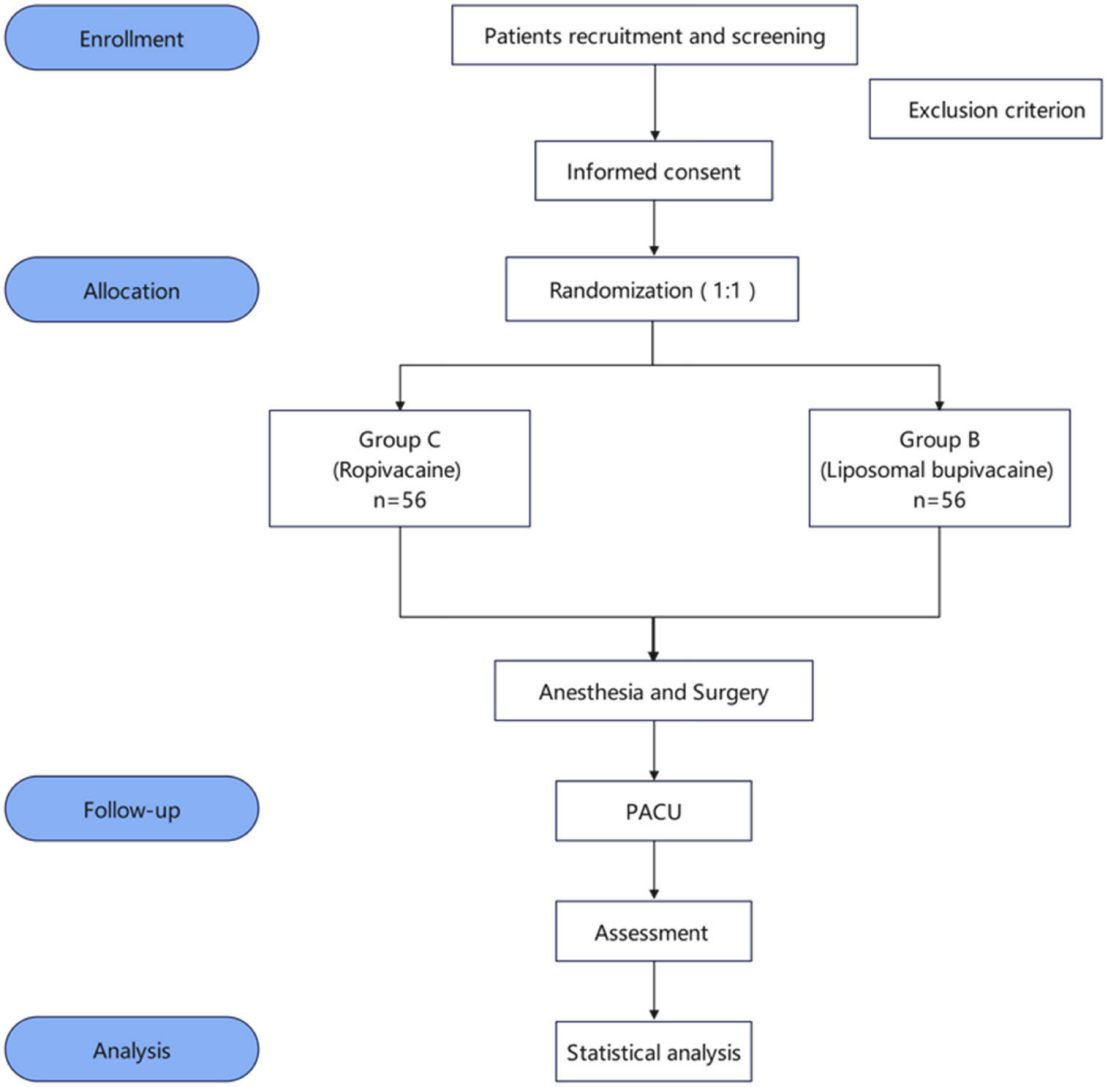
Flow diagram of this trial.

This trial is a prospective, single-center, randomized, double-blind clinical study designed in accordance with Standard Protocol Items: Recommendations for Interventional Trials reporting guidelines (32). Participants will be randomly assigned to either the liposomal bupivacaine (group B) or liposomal bupivacaine (group C) group in a 1:1 allocation ratio. The trial will be conducted at Shanghai General Hospital, China, in October 2024. The schedules for enrollment, allocation, and assessment are shown in Table 1. A flow diagram of this trial is shown in Figure 1.

**TABLE 1:** The schedule of enrollment, allocation and assessments.

	Enrollment	Allocation	Post operation				Close-out
Timepoint	−1 day	0 day	PACU	24 h	48 h	72 h	
**Enrollment:**							
Eligibility screen	×						
Informed consent	×						
Allocation		×					
**Intervention:**							
Ropivacaine group (group C)		×					
Liposomal bupivacaine group (group B)		×					
**Assessment:**							
Base line	×	×					
MAP and HR		×					
Sufentanil dose		×	×				
Use of other analgesics		×	×	×	×	×	
Pain VAS			×	×	×	×	
PONV score				×	×	×	
Extubation time and PACU stay			×				
QoR-15						×	
Complications			×	×	×	×	
Adverse events			×	×	×	×	
Costs							×

HR, heart rate; MAP, mean arterial pressure; VAS, visual analog scale; PONV, postoperative nausea and vomiting; PACU, post-anesthesia care unit; QoR-15, quality of recovery-15; h, hour.

### 2.2 Objectives

The main objective of this trial is to implement preemptive analgesia through pre-incisional infiltration with bupivacaine liposomes. We aim to investigate the effects of this method on patients’ pain scores from 1 to 3 days postoperatively and to assess the patients’ reactions to skin incision, opioid consumption, and quality of recovery, with the ultimate goal of validating the superiority of pre-incisional infiltration with bupivacaine liposome in postoperative pain management in patients undergoing acoustic neuroma surgery.

### 2.3 Recruitment and ethics

This study has been approved by the Ethics Committee of Shanghai General Hospital and registered with the Chinese Clinical Trial Registry (ChiCTR2400090509; Principal investigator: Feng Chen; Registration Date: October 8, 2024). The trial protocol has been modified twice, with the final version being V3.0/20240715. This trial does not involve the collection of biological samples. Patients scheduled for acoustic neuroma surgery will be recruited and screened by the investigators 1 day prior to surgery. Those who meet the inclusion criteria will be scheduled for a subsequent interview, during which they will receive an informed consent discussion and will sign an enrollment form.

### 2.4 Eligibility criteria

#### 2.4.1 Inclusion criteria

(1)    Age >18 years
(2)    Scheduled for elective acoustic neuroma surgery
(3)    American Society of Anesthesiologists (ASA) physical status I-III
(4)    Inform the relevant contents of this clinical trial and sign the informed consent

#### 2.4.2 Exclusion criteria

(1)    Combination of severe cardiopulmonary, hepatic, or renal dysfunction
(2)    History of mental illness or current use of psychotropic medications
(3)    History of neurological diseases (e.g., cerebral infarction, transient ischemic attack, cerebral hemorrhage, Parkinson’s disease, intellectual disability, or craniocerebral injury).
(4)    Heart rate (HR) <50 bpm or prolonged QT interval on electrocardiogram (ECG)
(5)    ECG indicating pre-excitation syndrome or confirmed history thereof
(6)    Allergy to ropivacaine or bupivacaine liposome
(7)    Puncture site or systemic infection
(8)    History of craniotomy
(9)    Postoperative sedation for any reason.
(10)    Inability to understand visual analog scores (VAS)
(11)    Refusal to sign informed consent

#### 2.4.3 Withdrawal criteria

(1)    Delayed extubation after surgery or admission to the intensive care unit with endotracheal intubation
(2)    Need for second operation within the postoperative operation
(3)    Poor postoperative cognitive function within 72 h

### 2.5 Randomization and blinding

In this study, the method of randomization is block randomization. The block length is eight, and patients will be randomly assigned in a 1:1 ratio to either the liposomal bupivacaine or ropivacaine group. After designing the serial numbers according to the blocks, the numbers will be arranged in ascending order both within and across the blocks. An envelope is encoded for each number. The allocation sequences will be placed in sequentially numbered opaque sealed envelopes. On the day of surgery, a nurse will open the envelope to prepare the solution according to the protocol. The prepared solution will be placed in a light-protected syringe and administered by a neurosurgeon. Participant recruitment will be conducted by an anesthesiologist who will not be involved in data collection and analysis. Upon unblinding, the envelopes will be opened in a strict numerical order. The envelopes will be managed by a certified nurse who will not be involved in any other aspect of the study, beyond managing the envelopes, unblinding the participants, and dispensing medication. Given the single type of surgery and the single-center nature of the study, stratification is deemed impractical. Additionally, the surgical procedure for acoustic neuroma surgery is standardized, with patients usually positioned laterally, and the surgical approach is typically through the sigmoid sinus. A relatively fixed incision area ensures better intervention consistency.

The participants, investigators, and evaluators will be blinded to the study procedure. Owning to the distinct properties of the two drugs, the bupivacaine liposome injection solution appears as a white suspension, whereas the ropivacaine injection solution is a colorless transparent liquid-dark; hence, a light-resistant syringe will be used during dispensing to maintain blinding. This prevents the identification of the agent from the outside. The surgeon is responsible only for scalp injections and will not participate in screening, enrollment, surgical anesthesia, or follow-up. Researchers involved in screening, enrollment, surgical anesthesia, and follow-up, including the patients themselves, will be unaware of the grouping.

### 2.6 Intervention description

The intervention in this study involves preoperative infiltration of the patient’s scalp incision by the neurosurgeon using a 22-gauge needle under sterile conditions. Neurosurgeons will prepare separate local anesthetics before surgery in the operating room. In Group B, 20 mL of the original bupivacaine liposome solution will be used and in Group C, 0.75% ropivacaine (4 mL) will be diluted to a total volume of 20 mL with normal saline.

### 2.7 Anesthesia management

An identical anesthesia management technique will be used for all patients. Upon admission to the operating room, continuous monitoring of the patient’s vital signs will commence, including ECG, non-invasive blood pressure, HR, and oxygen saturation. Subsequently, peripheral venous access will be established, and invasive blood pressure monitoring will be initiated via femoral artery cannulation under local anesthesia. The nasopharyngeal temperature will also be monitored following intubation. Preoxygenation is a critical component of anesthesia induction. This involves the administration of intravenous propofol at a dosage of 2 mg/kg, sufentanil at 0.25 μg/kg, and rocuronium at 0.6 mg/kg. Following endotracheal intubation, we will utilize a combination of intravenous and inhalational anesthesia. Desflurane will be set at 0.4 MAC, and propofol will be maintained between 2–4 μg/mL to ensure that the bispectral index values remain within the range of 50–60. This parameter will serve as an indicator for monitoring the depth of anesthesia. The ventilation mode is configured for volume control ventilation, with adjustable parameters that include an FiO_2_ level of 60%, a tidal volume (VT) ranging from 6 to 8 mL/kg, and a respiratory rate set between 10 and 15 breaths per minute to maintain end-tidal carbon dioxide levels within the range of 35–45 mmHg. Given the extended duration of acoustic neuroma surgery, intraoperative monitoring of neurological function is imperative. Given that the use of muscle relaxants may interfere with this monitoring process, no additional muscle relaxants will be administered during the procedure. Before the skin incision, 5–10 μg of sufentanil will be administered to the patient based on their weight and current circulatory status. A routine dose of 10 μg of sufentanil will be given to the patient 30 min prior to the conclusion of surgery. Palonosetron (0.25 mg) will be administered prior to skin closure to prevent nausea and vomiting. Continuous infusion of remifentanil (0.15–0.3 μg/kg/min) will be utilized for intraoperative analgesia. This infusion will be gradually tapered off and discontinued 30 min before the end of surgery. The infusion rate of remifentanil will be adjusted to maintain mean arterial pressure (MAP) and HR within ±20% of the baseline values. During recovery, sugammadex will be employed for muscle relaxant antagonism. Intraoperative hypotension will be managed with ephedrine (6 mg) or epinephrine (40 μg). The treatment of bradycardia (<50 bpm) will involve the administration of atropine in a bolus dose of 0.01 mg/kg. If sympathetic excitation occurs, in addition to deepening anesthesia, nimodipine or esmolol may be used to maintain blood pressure and HR within normal ranges. Following the procedure, the patient will be transferred to the post-anesthesia care unit (PACU), where both anesthesiologists and nurse anesthetists will be responsible for the extubation and monitoring of the patient’s condition. The extubation criteria include achieving a VT of 5 mL/kg and a respiratory rate of 12 breaths per min, while ensuring that the patient awakens with a clear swallowing reflex. Additionally, oxygen saturation must remain at or above 95% after breathing room air for 5 min.

### 2.8 Follow-up

The follow-up period will last for 3 days after surgery. Assessments will be conducted at different time points, including pain VAS score, analgesic drug use and frequency, PONV score, postoperative rehabilitation quality score, postoperative hospital stay duration, and cost. Any perioperative adverse reactions related to the surgical procedure, anesthesia, or medication administration will be documented.

### 2.9 Pain management

Multimodal analgesic program:

(1)    Preemptive analgesia: preoperative pre-incisional infiltration anesthesia.
(2)    Intraoperative: 10 ug of sufentanil and 50 mg of flurbiprofen will be administered before the end of surgery.
(3)    Resuscitation room: pain control after recovery will be managed by the anesthesiologist in the resuscitation room. If the pain score is 4 or higher, 50 mg flurbiprofen will be given intravenously.
(4)    Postoperative: oral celecoxib 200 mg twice daily will be chosen for postoperative analgesia. If the pain score is >4, 100 mg tramadol will be administered intramuscularly.

## 3 Outcome measures

### 3.1 Baseline data

Demographic characteristics include body mass index (BMI), age, sex, ASA status, acoustic neuroma size, and comorbidities. Surgical and anesthetic characteristics, including incision length, local anesthetic dosage, duration of surgery and anesthesia, intraoperative analgesic dosages (sufentanil and remifentanil), and hemodynamic parameters will be measured at five time points during the perioperative period.

### 3.2 Primary outcome

The primary outcome is the 24 h postoperative pain VAS scores.

### 3.3 Secondary outcome

(1)    Postoperative pain VAS scores: pain VAS scores at 48 h and 72 h postoperatively.
(2)    Changes in vital signs: the MAP and HR at five time points: T1 (before anesthesia), T2 (at incision), T3 (during drilling of the skull), T4 (at skin closure), and T5 (at the end of the operation).
(3)    Intraoperative and postoperative analgesic use: intraoperative and resuscitation room opioid use, postoperative opioid use on days 1, 2, and 3, and the number and dosage of celecoxib administered will recorded. Data will be statistically converted to morphine doses.
(4)    Incidence of PONV: the preoperative Apfel risk score includes four items: female sex, history of PONV or motion sickness, non-smoking status, and history of opioid use. The incidence of PONV in patients with 0, 1, 2, 3, and 4 risk factors is 10, 21, 39, 61, and 78%, respectively. The Alfel risk score ranges from 0 to 4, with higher scores indicating a higher risk. PONV will be assessed using the VAS scale (0–10), where 0 indicates no nausea; 1–4 indicates mild nausea; 5–6 indicates moderate nausea; and 7–10 indicates severe nausea.
(5)    Extubation time and PACU stay: time to extubation and duration of stay in the PACU after surgery.
(6)    Length of postoperative hospitalization and costs: duration of postoperative hospital stay and associated costs during hospitalization.
(7)    Postoperative rehabilitation quality assessment: the 15-item Quality of Recovery Scale will be used to assess postoperative rehabilitation quality. On the third day after surgery or prior to discharge, patients will be asked to complete a 15-question questionnaire (Table 2). Response options range from 0 to 10 points, with 0 indicating poor condition and 10 indicating excellent condition.
(8)    Other postoperative complications: incidence and nature of other postoperative complications.

**TABLE 2:** 15-item quality of recovery scale (QoR-15).

Question	Score (0–10)
1. Do you feel that your breathing is comfortable?	
2. Is your appetite good?	
3. Are you able to rest sufficiently and feel energetic as a result?	
4. How is your sleep quality?	
5. Are you able to take care of your personal hygiene independently?	
6. Are you able to have normal conversations with family and friends?	
7. Do you feel supported and cared for by the medical staff?	
8. Are you able to engage in normal work or household activities?	
9. Do you feel comfortable and able to manage your emotions?	
10. Do you feel generally happy?	
11. Do you have severe pain that affects your sleep?	
12. Do you have severe pain that is unbearable?	
13. Do you have nausea or vomiting?	
14. Do you feel tense or anxious?	
15. Do you feel sad or depressed?	

## 4 Statistical methods and sample size

Data analyses will be performed using IBM SPSS Statistics version 20. In addition to the primary analysis, multivariable linear regression will be performed to adjust the primary outcome (24-h VAS score) for potential confounding variables, including age, sex, BMI, ASA physical status, duration of surgery, and tumor size. Exploratory subgroup analyses based on these factors will be conducted if the sample size permits. Normally distributed data will be reported as mean ± standard deviation (mean ± SD), while non-normally distributed data will be presented as median (N) with interquartile range. Categorical data will be shown as percentages (%). Pain scores at different time points will be compared using independent sample *t*-tests, and group comparisons will be analyzed using repeated-measures chi-square analysis. Other metrics will be assessed using χ^2^ tests, *t*-tests, or Mann–Whitney U tests as appropriate. A *p*-value < 0.05 will be considered statistically significant.

Based on prior literature (33), the parameters are set as follows: β = 0.2, α = 0.05, *R* = 1, μ1 = 2.9, μ2 = 3.7, and σ = 1.4. The sample size is calculated using PASS 15.0 statistical software, resulting in 50 effective cases per group. Considering the potential sample loss of 10% and the block size, 112 patients will be enrolled in this study.

## 5 Data collection

The intraoperative anesthesiologists and postoperative follow-up researchers will be blinded to the study design and group allocation. This ensures unbiased data collection of intraoperative vital signs and postoperative pain. This approach minimizes the potential reporting errors. A detailed overview of the collected data is shown in Table 1.

## 6 Data monitoring and management

The study will establish a Data Safety Monitoring Board (DSMB) comprising 10 senior anesthesiologists, surgeons, and statistical experts, each with over 20 years of clinical experience. The DSMB will periodically evaluate the safety of the study, verify the authenticity and integrity of the data, and assess the reliability of implementation procedures. The trial will undergo an annual review by the Ethics Review Committee of Shanghai First People’s Hospital. During this period, the trial will be placed on hold, and participants will not be involved in the review process. Following the review, the trial will either resume or remain suspended based on the audit outcomes. All participants will sign a confidentiality agreement to ensure individual accountability for the accuracy of the data they provide, to maintain data confidentiality, and to protect the privacy of their personal information. All participant information and data will be meticulously recorded in a case report form. Participants who discontinue or deviate from the intervention protocols will not be replaced. Their data will be retained until study completion, except for those who cannot complete the primary outcome assessment. Upon collection and organization, the data will be securely transferred to the principal investigator for storage throughout the duration of the study. Access to the data will be strictly limited to the principal investigator. Other researchers who require access to study data may contact the principal investigator after the completion of the trial to request access, subject to approval and appropriate data-sharing agreements.

## 7 Dissemination plans

The results will be published in peer-reviewed journals and presented at relevant scientific conferences. They will also be shared with stakeholders to guide future research.

## 8 Harms

The treatment measures in this study involve the use of local anesthetics for incisional infiltration. There is a risk of local anesthetics entering blood vessels. To mitigate this risk, the syringe is routinely aspirated before the procedure to confirm the absence of blood before proceeding with incision infiltration. However, the concentration of the drugs used in this trial is low, and the dose is small. The physicians performing the procedures are highly skilled, and the administered dose is well below the threshold that can cause local anesthetic toxicity. In the event of accidental symptoms of local anesthetic toxicity or circulatory fluctuations, immediate symptomatic treatment will be administered, and adverse events will be recorded. Adverse events related to study interventions will be treated for free.

## 9 Discussion

In this randomized controlled trial, we will compare the effects of local scalp infiltration anesthesia with ropivacaine and liposomal bupivacaine on postoperative pain in patients undergoing acoustic neuroma surgery. To our knowledge, liposomal bupivacaine has been commercially available for a relatively short time; thus, there are almost no studies on its use for scalp incision infiltration in craniotomy surgery. Liposomal bupivacaine is a novel anesthetic agent consisting of multivesicular bupivacaine liposomes. It degrades slowly through internal fusion and fission, allowing a single dose to provide local postoperative analgesia for up to 72 h. This significantly extends the duration of action of the local anesthetic and fills the gap in pain control that exists with traditional local anesthetics (34, 35). Ropivacaine is a local infiltration anesthetic commonly used in clinical practice. The 0.15% ropivacaine concentration used in this study carries a relatively low risk of local anesthetic toxicity.

Our study has some limitations. First, this is a single-center, single-disease study. However, as a large, comprehensive medical institution, our neurosurgery department is a key specialty with extensive experience in acoustic neuroma surgeries, and our patients come from a wide range of regions. Therefore, our findings may have a significant guiding value. Second, we only compare the postoperative analgesic effects of the two local anesthetics and conduct a single-concentration comparison without setting up multiple groups to determine the optimal drug concentration for postoperative analgesia. Future clinical trials should explore the optimal liposomal bupivacaine concentrations. Finally, patients undergoing neurosurgery often exhibit higher levels of anxiety and depression because of the disease itself (symptoms such as dizziness and tinnitus) and concerns about surgery. These factors can influence postoperative pain perception and recovery (36). However, owning to the lack of specialized preoperative psychological assessments, it is difficult to control these variables effectively. Therefore, future research should explore ways to improve patients’ anxiety and depression through preoperative psychological interventions, thereby reducing postoperative pain.

In conclusion, the effective control of postoperative pain is of great significance in patients with acoustic neuroma. If our results demonstrate that pre-incisional infiltration with liposomal bupivacaine can significantly reduce the use of analgesics, we will be able to offer more effective pain management options for these patients.
